# 1387. Vaccination against respiratory pathogens during pretravel consultations at US Global TravEpiNet Sites: interventions and missed opportunities.

**DOI:** 10.1093/ofid/ofad500.1224

**Published:** 2023-11-27

**Authors:** Loukas Kakoullis, Sowmya R Rao, Edward T Ryan, Regina C LaRocque, Lin H Chen

**Affiliations:** Mount Auburn Hospital, Watertown, Massachusetts; Boston University, Boston, Massachusetts; Massachusetts General Hospital, Boston, MA; Massachusetts General Hospital, Boston, MA; Mount Auburn Hospital, Watertown, Massachusetts

## Abstract

**Background:**

We sought to evaluate the contribution of Global TravEpiNet (GTEN) sites in providing influenza and pneumococcal vaccinations during pretravel visits.

**Methods:**

We analyzed prospectively-collected data from 31 GTEN sites between 07/01/2012 -06/31/2022 and included demographics, travel-related characteristics and vaccines administered at the visit. Vaccine eligibility was based on CDC recommendations. Influenza seasonality at the destination was determined based on WHO data^1^. We conducted descriptive analyses on vaccinations and reasons for non-administration (SAS v9.4; Cary, NC).

**Results:**

The 10-year study period included 116,865 pretravel visits during which clinics administered 1,732 pneumococcal and 15,658 influenza vaccines (Table 1) and referred an additional 3,588 patients to their PCPs for pneumococcal and 6,955 for influenza vaccination.

Of travelers eligible for pneumococcal vaccination, 49.9% were not recognized as having indications for vaccination. In the case of influenza, the vaccine was not offered to 14.3% of eligible travelers due to unavailability (6.9%) or because the provider did not consider it to be indicated based on the itinerary (7.4%).

Among 66,056 travelers with an itinerary falling within an area with active influenza transmission, the vaccine was unavailable for 10% and not considered indicated in 9.5% (Figure 1). These issues occurred mostly during the summer months in the USA. Specifically, 89.8% of travelers for whom there was no available vaccine, and 83.4% of travelers not recognized as eligible for the vaccine, were traveling between April and September.

**Figure:**
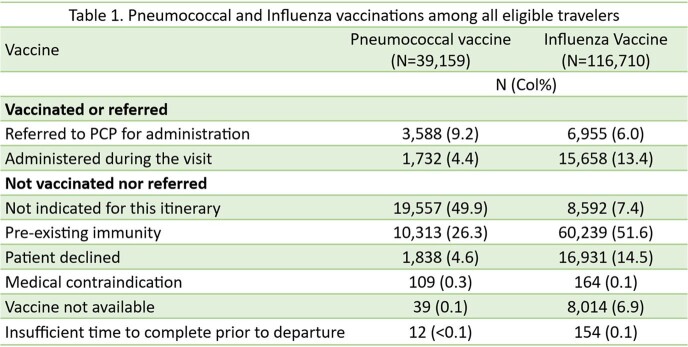

**Figure:**
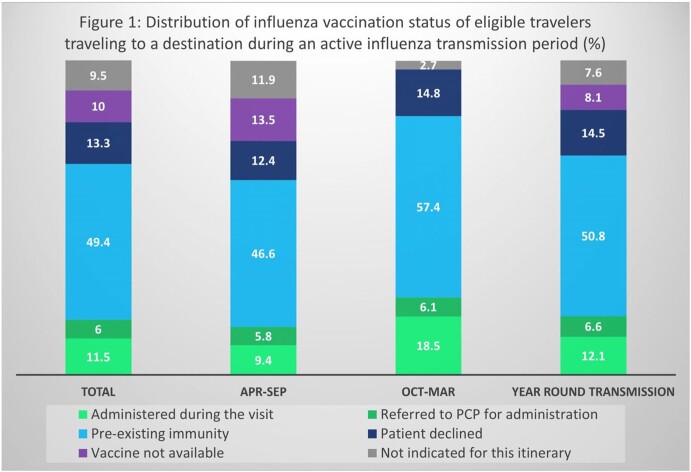

**Conclusion:**

GTEN clinics provide routine vaccinations against respiratory pathogens, but some missed opportunities are identified. GTEN providers often referred patients to their PCPs for pneumococcal vaccination, perhaps reflecting issues of insurance coverage. The influenza vaccine expiration dates lead to lack of the vaccine for almost 3 months, during summer in the USA. Finally, persons traveling between April and September or to areas with year round transmission may not be recognized to have potential influenza exposure, despite traveling to destinations with active influenza transmission.

References:

1. WHO Global Influenza Programme. https://www.who.int/tools/flunet

**Disclosures:**

**Lin H. Chen, MD**, Merck: Honoraria|Sanofi: Honoraria|Shoreland Inc: Advisor/Consultant|Valneva: Advisor/Consultant

